# Functional polymorphism of the *NFKB1 *gene promoter is related to the risk of dilated cardiomyopathy

**DOI:** 10.1186/1471-2350-10-47

**Published:** 2009-05-31

**Authors:** Bin Zhou, Li Rao, Ying Peng, Yanyun Wang, Yi Li, Linbo Gao, Yu Chen, Hui Xue, Yaping Song, Miao Liao, Lin Zhang

**Affiliations:** 1 Laboratory of Molecular Translational Medicine, West China Second University Hospital, Sichuan University, Chengdu 610041, PR China; 2 Department of Cardiology, West China Hospital of Sichuan University, Chengdu 610041, PR China; 3 Department of Immunology, West China School of Preclinical and Forensic Medicine, Sichuan University, Chengdu 610041, PR China; 4 Department of Forensic Biology, West China School of Preclinical and Forensic Medicine, Sichuan University, Chengdu 610041, PR China

## Abstract

**Background:**

Previous studies in experimental and human heart failure showed that nuclear factor kappa B (NF-κB) is chronically activated in cardiac myocytes, suggesting an important involvement of NF-κB in the cardiac remodeling process. A common insertion/deletion (-94 insertion/deletion ATTG, rs28362491) located between two putative key promoter regulatory elements in the *NFKB1 *gene was identified which seems to be the first potential functional *NFKB1 *genetic variation. The main goal of the present investigation was to investigate the *NFKB1 *-94 insertion/deletion ATTG polymorphism in relation to risk of dilated cardiomyopathy (DCM).

**Methods:**

A total of 177 DCM patients and 203 control subjects were successfully investigated. The *NFKB1 *-94 insertion/deletion ATTG polymorphism was genotyped by using PCR-PAGE.

**Results:**

Genotype frequency of *NFKB1 *-94 insertion/deletion ATTG polymorphism in DCM patients was significantly different from that in control subjects (*P *= 0.015) and the ATTG_2 _carrier (ATTG_1_/ATTG_2 _+ ATTG_2_/ATTG_2_) was susceptible to DCM.

**Conclusion:**

Our data suggested that *NFKB1 *-94 insertion/deletion ATTG polymorphism is associated with DCM.

## Background

Dilated cardiomyopathy (DCM), characterized by left ventricular dilation and systolic dysfunction, is the most common form of heart muscle disease, comprising 60% of the cases of identified cardiomyopathy [1]. This disorder is clinically heterogeneous, ranging from affected individuals with clinical presentations of severe symptoms, including heart failure and sudden death, and asymptomatic individuals. The clinical course of DCM, almost regardless of the underlying cause, may be progressive, with roughly 50% of individuals reported to die within 5 years of diagnosis without transplantation [1,2]. Although longer survival has been accomplished recently with improved medical therapies and interventions, early examinations are necessary to improve the DCM prognosis. The etiology of DCM is multifactorial and many different clinical conditions can lead to phenotype of DCM. It has become evident that genetic factors play an important role in the etiology and pathogenesis of DCM. To date, DCM-associated mutations in many different genes have been reported, but still these mutations explain only a minority of the etiology of DCM [3]. Some susceptibility genes have been shown to be associated with an increased risk of developing a DCM. These include HLA-DQA1*0501, HLA-DRB1*1401, exon 8 C/T of Endothelin receptor A, Leu10Pro of TGF-beta1, G994T of PAF acetyl hydrolase, MMP-3 5A/6A, and so on [4-6]. With more knowledge about susceptibility genes and pathways involved in DCM, strategies may emerge to reduce myocyte death, and diminish myocardial fibrosis, processes that ultimately cause the heart fail.

Clinical observations and experimental studies have demonstrated that left ventricular (LV) remodeling and dilation occurs with the progression of end-stage LV failure. It has been reported that in experimental and human heart failure, nuclear factor kappa B (NF-κB) is chronically activated in cardiac myocytes, suggesting an important involvement of NF-κB in the cardiac remodeling process [7]. NF-κB is a redox-sensitive transcription factor regulating a battery of inflammatory genes and it has been implicated as important for initiation and progression of pathogenesis of many autoimmune and inflammatory diseases [8-10]. Numerous lines of investigation suggest that NF-κB could promote tumorigenesis [11]. Cardiac-specific expression of tumor necrosis factor (TNF) has previously been shown to produce DCM, presumably through intact TNF-related apoptosis [12]. In endothelin-1 deficient hearts NF-κB activity decreased, resulting in diminution of downstream inhibitors of TNF signaling [13]. It is apparent that the role of NF-κB in the regulation of cardiomyocyte viability is multidimensional and might contribute to the development of DCM.

A common insertion/deletion polymorphism (-94 insertion/deletion ATTG, rs28362491) located between two putative key promoter regulatory elements in the *NFKB1 *gene was identified which seems to be the first potential functional *NFKB1 *genetic variation. The presence of a 4 base pair (bp) deletion resulted in the loss of binding to nuclear proteins, leading to reduced promoter activity [14]. A research has shown that the deletion was associated with an increased risk for an inflammatory intestinal disorder-ulcerative colitis (US), but subsequently other study failed to replicate this association [14-19]. Furthermore significant associations of this polymorphism with other disease entities (type 1 diabetes, oral squamous cell carcinoma, colorectal cancer, and melanoma) have been reported [20-23]. However, its association with DCM is still unclear. The main goal of the present investigation was to determine the possible susceptibility of *NFKB1 *-94 insertion/deletion ATTG polymorphism on the occurrence of DCM.

## Methods

### Subjects

This study was approved by the hospital ethics committee and all subjects gave written informed consent to participate. One hundred and seventy seven unrelated DCM patients between September 2002 and March 2008 were enrolled in this study. The diagnosis of DCM was made in accordance with the revised criteria established by the 1995 World Health Organization/International Society and Federation of Cardiology Task Force on the Classification of Cardiomyopathy (DCM group) [2] The control group consisted of 203 healthy subjects from a routine health survey. All subjects were Han population living in Sichuan province of southwest China. Patients with a history of hypertension, coronary heart disease, cardiac valve disease, tachyarrhythmia, heavy alcohol intake, acute viral myocarditis, systemic diseases of putative autoimmune origin, or skeletal myopathies were intentionally excluded.

### Determination of genotypes

Genomic DNA of each individual was extracted from 200 μl EDTA-anticoagulated peripheral blood samples by a DNA isolation kit from Bioteke (Peking, China) and the procedure was performed according to instruction manual. The polymerase chain reaction (PCR)-polyacrylamide gel electrophoresis (PAGE) method was used to genotype the -94 insertion/deletion ATTG polymorphisms of *NFKB1*. DNA fragments containing the polymorphism were amplified and the primer sequences were: F 5'-tggaccgcatgactctatca-3', R 5'-ggctctggcttcctagcag-3'. PCR reaction was performed in a total volume of 25 μl, including 2.5 μl 10× PCR buffer, 1.5 mmol/L MgCl_2_, 0.15 mmol/L dNTPs, 0.5 μmol/L each primer, 100 ng of genomic DNA and 1 U of *Tag *DNA polymerase. The PCR conditions were 94°C for 4 min, followed by 32 cycles of 30 s at 94°C, 30 s at 64°C and 30 s at 72°C, with a final elongation at 72°C for 10 min. 3 μl PCR products were separated by a 6% polyacrylamide gel and staining with 1.5 g/L argent nitrate. Allele (ATTG)_1 _yield a 154 bp band and allele (ATTG)_2 _yield a 158 bp band. About 20% of the samples were randomly selected to perform the repeated assays and the results were 100% concordant.

### Statistical analysis

All data analyses were carried out using SPSS 13.0 statistical software. Allelic and genotype frequencies of *NFKB1 *gene -94 insertion/deletion ATTG polymorphism were obtained by directed counting and Hardy-Weinberg equilibrium were evaluated by chi-square test. Odds ratio (OR) and respective 95% confidence intervals were reported to evaluate the effects of any difference between allelic and genotypes distribution. Probability values of 0.05 or less were regarded as statistically significant in DCM patients compared to healthy controls.

## Results

Genotype distributions had no deviation from Hardy-Weinberg equilibrium both in patients and controls. Differences in allelic and genotype distribution of *NFKB1 *gene -94 insertion/deletion ATTG polymorphism were tested between DCM patients and controls, and observed differences are presented in Table 1.

**Table 1:** The allelic and genotype distributions of *NFKB1 *polymorphism among patients and controls

Variables		Patients(n = 177)	Controls(n = 203)	*p*	OR	95% CI
*NFKB1*-94 genotype	ATTG_1_/ATTG_1_	18(10.2)	42(20.7)	-	1	-
	ATTG_1_/ATTG_2_	95(53.7)	90(44.3)	**0.004**	**2.463**	**1.321–4.592**
	ATTG_2_/ATTG_2_	64(36.1)	71(35.0)	**0.023**	**2.103**	**1.101–4.018**
	ATTG_1_/ATTG_2_+ATTG_2_/ATTG_2 _versus ATTG_1_/ATTG_1_			**0.005**	**2.304**	**1.272–4.174**
	ATTG_1_/ATTG_1_+ATTG_1_/ATTG_2 _versus ATTG_2_/ATTG_2_			0.810	1.053	0.691–1.604
	
*NFKB1*-94 allele	ATTG_1_	132(37.3)	174(42.9)	0.118	1.261	0.942–1.688
	ATTG_2_	222(62.7)	232(57.1)			

Note: Bold-faced values indicate significant difference at the 5% level.

The overall genotype frequency of DCM patients was significantly different from that of controls. The frequency for ATTG_1_/ATTG_1 _genotype was slightly overrepresented in controls (*P *= 0.004, OR = 2.463, 95%CI = 1.321–4.592 for ATTG_1_/ATTG_1 _vs. ATTG_1_/ATTG_2 _comparison, and *P *= 0.023, OR = 2.103, 95%CI = 1.101–4.018 for ATTG_1_/ATTG_1 _vs. ATTG_2_/ATTG_2 _comparison, respectively). Furthermore, the *P *value and OR were 0.005 and 2.304, respectively, ATTG_1_/ATTG_2 _+ ATTG_2_/ATTG_2 _vs. ATTG_1_/ATTG_1 _comparison, indicating that ATTG_2 _carrier (ATTG_1_/ATTG_2 _+ ATTG_2_/ATTG_2_) was susceptible to DCM. The frequency of allele ATTG_2 _in DCM patients was higher than that in control subjects (62.7% vs. 57.1%), but is not statistically significant (*P *= 0.118).

## Discussion

Genetic factors are known to play an important role in the etiology of DCM. The first DCM-associated mutation, in the dystrophin gene, was described in 1993 [24,25]. The genetic of DCM have been under intensive investigation lately and the knowledge on the genetic basis of DCM has increased rapidly. To date, DCM-associated mutations in many different genes with subsequent alterations in protein structure have been reported, but these mutations explain only a minority of the etiology of DCM [3]. Some susceptibility genes have also been shown to be associated with an increased risk of developing a DCM.

NF-κB was discovered by Baltimore and coworkers in 1986 as a *f*actor in the *n*ucleus of *B *cells that binds to the enhancer of the *k*appa light chain of immunoglobulin [11,26]. The transcription of many genes for immune response, cell adhesion, differentiation, proliferation, angiogenesis and apoptosis are regulated by NF-κB. It is just this characteristic that makes NF-κB the crucial point of convergence of a number of stimuli that can influence different aspects of cellular homeostasis [27]. Inappropriate activation of NF-κB can mediate inflammation and tumorigenesis. How NF-κB activation mediates tumorigenesis and inflammation has been widely studied during the past decade. Most inflammatory agents mediate their effects through the activation of NF-κB and it is suppressed by anti-inflammatory agents. Most carcinogens and tumor promoters activated NF-κB, whereas chemopreventive agents suppress it [11]. NF-κB is rarely found to be constitutively active in normal cells except for proliferating T cells, B cells, thymocytes, monocytes, and astrocytes, while it is constitutively active in most tumor cell lines [11,23,27]. However, the proposal that NF-κB leads to the onset of cancer has been changed by the evidence that, for skin cancer, NF-κB activation has been postulated as a safeguard against cancer [27,28]. Further studies could help to figure out the molecular mechanisms that dictate the pro-oncogenic or anti-oncogenic activity of NF-κB.

The -94 insertion/deletion ATTG polymorphism was identified in a study sequenced the *NFKB1 *promoter in 10 inflammatory bowel disease and 2 controls, and the ATTG_1 _allele was more frequent in ulcerative colitis than that in controls in the following study. The in vitro promoter expression studies suggest that the ATTG_1 _allele may result in relatively decreased *NFKB1 *message and hence decreased p50/p105 NF-κB protein production [14]. The association between -94 insertion/deletion ATTG polymorphism and susceptibility to ulcerative colitis was confirmed in another study, but data were inconsistent and this association could not be replicated in different population [16,17,29].

Given the considerable important role of NF-κB pathway involved in initiation and progression of pathogenesis in disease, we investigated the association between -94 insertion/deletion ATTG polymorphism and susceptibility to DCM. The present study shows that the allelic frequency for *NFKB1 *gene -94 insertion/deletion ATTG polymorphism in DCM patients is not significantly different from that of controls. However, the genotype frequency distribution in DCM patients was significantly different from that of controls. The frequency of the ATTG_1_/ATTG_1 _genotype of *NFKB1 *gene -94 insertion/deletion ATTG polymorphism in controls was significantly higher that in DCM patients. We conducted comparison between ATTG_1_/ATTG_1 _and (ATTG_1_/ATTG_2_+ATTG_2_/ATTG_2_) in DCM patients and controls, and further significant difference was observed (*p *= 0.005). These results indicated that ATTG_2 _carrier (ATTG_1_/ATTG_2 _+ ATTG_2_/ATTG_2_) was susceptible to DCM. The allelic distribution between DCM patients and controls was different, although not statistically significant (p = 0.118), and the frequency for ATTG_2 _allele in DCM patients is higher than that in controls, although not statistically significant, also indicating that ATTG_2 _allele might be a risk factor for the susceptibility to DCM.

The role of NF-κB in heart has been extensively studied by many authors [10]. Its role in the regulation of cardiomyocyte viability is multidimensional, because it was believed to activate cell-death pathway and it can also protect cells from death. In hearts subjected to in vivo infarction, NF-κB-activation is biphasic, peaking after 15 min and 3 h reperfusion, respectively. NF-κB might play a detrimental role during reperfusion and inhibition of leukocyte adhesion, cytokines, and chemokines which are regulated by NF-κB during reperfusion protects the heart against reperfusion injury [10,30]. A detrimental role of NF-κB-activation in cardiac allograft rejection has been suggested, as pharmacological inhibition of NF-κB prolongs survival of heterotopic transplants [31]. In atherosclerotic lesions the NF-κB-regulated inflammatory mediators such as cytokines, inducible NO synthase and leukocyte adhesion molecules have been detected, and mice deficient in NF-κB signaling exhibit reduced fatty-streak formation when fed a fatty diet [32,33]. Although the functional consequences are as yet undetermined, both systemic and cardiac activation of NF-κB have been found in unstable coronary syndromes. The present study shows that the genotype distribution of *NFKB1 *gene -94 insertion/deletion ATTG polymorphism in DCM patients is significantly different from that in control subjects, and (ATTG_1_/ATTG_2_+ATTG_2_/ATTG_2_) genotype frequency is higher in DCM patients than that in control subjects. Considering that the ATTG_1 _allele may result in decreased *NFKB1 *message and p50/p105 NF-κB protein production, a detrimental role of NF-κB-activation in the initiation and progression of pathogenesis in DCM can be presumed and much further work will be needed for a complete understanding of its mechanism. The main limitation of the present study is the relatively small size of the study population and the lack of replication of the significant association in a second independent cohort of DCM patients.

## Conclusion

From this study we conclude that the *NFKB1 *-94 insertion/deletion ATTG polymorphism is significantly associated with DCM. The genotype frequency of (ATTG_1_/ATTG_2_+ATTG_2_/ATTG_2_) is significantly overrepresented in DCM patients, indicating that ATTG_2 _carriers are associated with the increased risk of DCM. However, additional studies in a larger number of DCM patients and in different populations could help to establish the true significance of this association.

## Competing interests

The authors declare that they have no competing interests.

## Authors' contributions

BZ, LR and LZ conceived of the study, participated in its design, carried out most of the experiments and drafted the manuscript. YL, LG and YW participated in design of study and helped to draft the manuscript. YC, HX and YS performed sample collection and DNA extraction. YP and ML participated in genotyping. All authors have read and approved the final manuscript.

## Pre-publication history

The pre-publication history for this paper can be accessed here:


